# Users' Rating Predictions Using Collaborating Filtering Based on Users and Items Similarity Measures

**DOI:** 10.1155/2022/2347641

**Published:** 2022-07-08

**Authors:** Sofia Nudrat, Hikmat Ullah Khan, Saqib Iqbal, Mian Muhammad Talha, Fawaz Khaled Alarfaj, Naif Almusallam

**Affiliations:** ^1^Department of Computer Science, COMSATS University Islamabad, Wah Campus, Wah Cantt 47040, Rawalpindi, Pakistan; ^2^College of Engineering, Al Ain University, Al Ain, UAE; ^3^Department of Computer and Information Sciences, Imam Mohammad Ibn Saud Islamic University, Riyadh, Saudi Arabia

## Abstract

The social media has made the world a global world and we, in addition to, as part of physical society, are now part of the virtual society as well. There has been the generation of a large amount of information over the social web. By way of increasing online information, new opportunities emerged, and diverse issues have been raised, which have attracted researchers to address these research problems. In this current age, where online business and e-commerce are part of our daily lives, recommender systems (RSs) are very effective for information filtering. RSs play a significant role in our lives by assisting users in recommending items and services what they may be interesting in to purchase or avail. In this research work, our goal is to predict the users' ratings for various items, which are an active research area in collaborative filtering (CF). In this work, we have explored various similarity measures based on user-user and item-item rating predictions on different datasets by applying collaborative filtering approaches. The comparison of item-item and user-user CF algorithms such as user K-Nearest Neighbour using cosine; similarity, Pearson correlation as well as item-based K-NN using these measures with baseline approaches and matrix-based methods such as Matrix factorization (MF), biased MF, and factor wise MF has been carried out. For empirical-based comparison analysis, diverse approaches have been selected such as slope one, random, and global average, and it revealed that item-item K-NN using Pearson correlation has outperformed all other applied approaches. For the experiments, three real world and widely used datasets of MovieLens 1M, CiaoDVD, and MovieLens 100k have been used. The empirical-based results have been evaluated by using standard performance evaluation measures of RMSE and MAE.

## 1. Introduction

In current years, online social networking platforms such as Google, Facebook, YouTube, and Twitter have connected people closer to each other. These platforms offer a large amount of online content that makes it very difficult to find desirable or relevant information, and a user may find it difficult to make effective decisions. This problem is commonly referred as the information-overloaded problem. To overcome such a problem, different online social networking platforms provide personalized Recommendation Systems (RSs) [1]. In the present era, RSs are becoming an integral part of many social media platforms or networks and businesses [2]. These RSs give users recommendations on the base of feedback. The feedback in the form of textual reviews or numeric ratings is collected by users using social media sites, after purchasing some items or products. With the help of the nature of reviews, we can understand why customers/users give items with such reviews.

RSs are engines that indicate a particular range of items to a user according to their past experiences, priorities, interest, and preferences [3, 4]. These preferences help to filter the item or content. There are multiple types of RS in daily lives, such as RS for YouTube, Netflix, Amazon, and News aggregators, which help users to have a variety of items on their preferences and help users to save their time and effort. For example, they can get a recommendation for movies, music, food, news, books, and hotels, depending upon their interest [5]. One of the main issues of an RS is maintaining the user's interest and recommending the most related content to the user. According to the user's priorities, if the content has a similarity with his preferences, they can view it; otherwise, they can ignore it.

There are three most popular types of RSs based on the methods used to develop recommender systems such as Collaborative Filtering, Content-Based Filtering, and Hybrid Filtering. Content-Based Filtering (CBF) is among the most adopted filtering techniques in RSs. CBF technique recommends items based on the previously viewed content present in the user's profile. The main aim of CBF is to suggest the objects or contents that the user likes in the past. CBF performs very efficiently on the recommendation of newly loaded items in the system [5]. CBF prediction technique is a Domain-Dependent approach. It depends upon the metadata of the items. That is, a rich amount of item's description and a very well-maintained user profile is required for the recommendation task. It emphasizes more on the item's attribute analysis to develop accurate predictions. A user profile is not that important for CBF because it does not influence the predictions [6].

Collaborative Filtering (CF) makes the prediction quite accurately that the users will agree on their taste in the future as they did in the past [7]. CF Technique is an independent domain technique for prediction. Some content like movies and music that metadata cannot easily describe. Therefore, CF is the best technique to adopt [8]. CF predicts the item by collaborating with the same interest users. For example, users like “Action Movies” to watch and user B also has the same genre. Therefore, user A's action movies that have been overlooked and rated will be recommended to user B. If user B has not watched them yet. In CF, an Item-User Matrix comprising the user's preferences are used. This User-Item Matrix database then matches the users with similar interests and choices to make recommendations. This process is also named “Neighborhood” [9]. These recommendations are generated by calculating the resemblance between the user profiles [10].

Hybrid Filtering is the third approach used in developing RS, with different techniques of CBF or CF, or other algorithms when combined from Hybrid Filtering (HF). HF provides optimized solutions to avoid limitations found in CF and CBF pure recommendation systems [11]. The basic idea of HF is that a combination of different approaches can provide better and more accurate predictions in single applied methods. A combined model can reduce the weakness of a single technique [8, 12].

In current years, a huge volume of content is being shared by online users on daily basis. Due to such information explosion, the Internet users may find it difficult to make effective decisions. Thus, to overcome such problems, different social platforms provide personalized recommendations as a basic need for the users. In the personalized recommendation system items are recommended by observing the behaviour and interests of the users through their feedback in the form of ratings, or reviews. By considering users' ratings, prediction using collaborating filtering based on users and items similarity is carried out in this research.

The rating prediction has been the subject of several research studies. Existing research studies lack to compare item-item and user-user CF algorithms with all mentioned baseline approaches. So, In this research study, our aim is to predict the user-user or item-item rating prediction based on user or item similarity measures. To address this issue, we explore diverse types of RS algorithms related to CF for item-item as well as user-user similarity using diverse formulas such as cosine similarity and Pearson correlation and compare these methods with widely used matrix-based approaches such as MF, biased MF, and factorwise MF. In addition, the baseline approaches such as slope one, random, and global average will be used for comparative analysis. We applied these methods on three widely used datasets and compared them using performance evaluation measures, i.e., RMSE and MAE to compare results.

The main research contribution of this research study includes:

- We have explored that item-item-based CF approaches are more effective compared to user-user-based CF approaches for recommendation systems
- It has been figured out empirically that Pearson correlation-based CF approaches show better results compared to cosine similarity-based CF approaches
- The experiments have been performed and these CF based approaches have been compared with matrix factorization-based methods and other standard baseline methods; the Pearson correlation-based CF algorithms outperformed all other algorithms
- The experiments have been performed on three widely used datasets and the evaluation has been carried out using standard performance evaluation measures of RMSE and MAE

The remainder of the article is structured in the following manner. The state-of-the-art approach of RS is presented in Section 2.  Section 3 presents the problem statements, and Section 4 presents the methodology; Section 5 presents the experimental setup and performance measurements. The results and discussion are presented in Section 6.

## 2. Related Work

Let us discuss the previous literature review on recommender systems and RS techniques. To increase the accuracy of recommendation prediction, many approaches for rating predictions are designed to improve the cold start problem and alleviate the sparse data.

### 2.1. Collaborating Filtering-Based Approaches

In recent years, numerous new CF models have been introduced that compare their accuracy measures with the baseline methods or existing models. The most extensive and commonly applied approach in the recommender systems [13], are CF based methods. In the article, [14], we proposed a collaborative recommendation system with a deep learning method (DLCRS). This method improves the problem of lack of time sparsity, low ranked approximation, and shortage of meaningful signals. The article makes a comparative analysis between the existing methods and the proposed methods. Results show that proposed approaches give more appropriate results than the existing approaches. In the article, [15], we proposed a novel technique that combines similarity measurement based on ranking and similarity measurement based on structure. The author finds that CFs memory-based combined with similarity measurement shows appropriate accuracy prediction compared to CFs model-based.

The research article [16] proposed a similarity measure multifactor that captures the nonlinear and linear correlation between resulting users from extreme behavior. This method improves the sparse scenario. In the framework of probability matrix factorization and of probability matrix factorization, a fusion approach is given that simultaneously considers global rating information and multifactor similarity. For improving the sparse data optimization, the global rating process with users' local relations. These are required for extremely sparse data. The result shows that the proposed method improves the sparse data robustness and prediction results compared to the collaborative filtering matrix factorization model. The article [17] proposed a simple linear model named UserReg, based on the matrix factorization (MF) model. This model uses explicit feedback information for rating prediction that helps regularize users' profile representation, the proposed method applied to the MovieLens dataset, FilmTrust (https://guoguibing.github.io/librec/datasets.html Accessed on May 03, 2021), and Yelp (http://yelp.com/dataset-change/ Accessed on May 03, 2021.). The significant findings of the results showed that UserReg outperforms the baseline methods, as well as it performed comparatively very well compared to other computationally complex models. Moreover, the two robust baseline methods, SVD++ and Biased MF have not been compared with the current novel methods. If both these were compared, the result would favor these two powerful baseline methods.

In the article [18], the author solves the low data utilization in recommendation results and proposed the *α*-divergence based on an item similarity measure, which computes the rating with a density probability distribution and reduces curated case dependency. The method also influences the rating absolute number and on the computation of results, curated case proportion, which substantially increases the recommendation accuracy. The proposed method is applied to the MovieLens and FilmTrust datasets and shows efficient results.  Table 1 shows the comparison of previous work of CF.

### 2.2. Content-Based Approaches

CB recommender system is basically designed to recommend items and objects related to users' past preferences [19], and only the rated items features and target user ratings have been considered in the approaches of CB [20]. Machine learning different algorithms such as Support Vector Machine (SVM), and KNN has been used in CB recommender systems for implementation purpose. To make recommendations in the CB approach uses information, i.e., social media content. The fields preferred for the approaches of CB are books, movies, and music that can be available as a text [21]. Here, some of last year's approaches have been discussed.

In article [22], for online shopping, a semantic web mining method for a recommender system has been proposed. The method has two phases. Firstly, textual data preprocessing, the mixture of existing ontology and developed ontology is used. Then, by using the Naïve Bayes algorithm, the recommendation has been generated. The experimental result shows that this method increases 5.2% accuracy. In the article [23], the traditional recommender system context has focused on using Goodreads book and MovieLens 20M datasets. The authors can use a gradient boosting machine (XGBoost) by using content-based and link stream features and present a content-based solely solution. For examination of this model generates a full state-of--of-the--the-art recommender system algorithm. The result shows better accuracy. In the present time, online discussions are represented by e-learning systems as a standard for combined learning, which supports the information exchanging and knowledge sharing between learners. The fast development of such mediums has created an issue to find relevant or interesting information. By noticing this problem, the article [24], proposed a new design for e-learning that recommends relevant or interesting information in an online e-learning discussion environment for learners. The design is based on the semantic CB recommender system and negative learner's ratings. The model exploits negative learners' ratings to achieve a key point in e-learning RSs, which confirms that the items recommended by the recent learners. The experiment was conducted between 25 students. The result shows that this technique gives better results compared to other approaches having similar models.

Group recommender systems have developed a result to recommend useful, suitable, and interesting items to groups of people rather than to individuals. Within this growing environment, such methods have pushed for the introduction of novel recommendation approaches, with the CF paradigm at the foundation of the recommender system. However, CB gives many limitations and drawbacks in these situations such as for several users and item co-occurrence as well as lots of rating values. To overcome this issue, the article [25], we proposed a CB group recommendation system (CB-GRS), and the article analyses and discusses three definite models for the building of CB-GRS: (1) individual ranking and aggregation recommendation, (2) user item matching and aggregation recommendation, and (3) aggregation of the user profile. This research work gives a hybrid CB-GRS that combines methods (2) and (3) and integration function and weighting features.  Table 2 shows the existing work of CBF.

The users of social media platforms may influence their thoughts, ideas, and views on a variety of topics while also generating the information that can be used to better understand people's reactions to certain items. A similar novel research study [26] has been conducted to detect the category of users reviews on social media about restaurants. The proposed method includes next word, next sequence, and pattern prediction algorithms are combined with Aspect Category Detection to create a convolutional attention-based bidirectional modified LSTM. The result of the study favours the proposed technique as it outperforms all the compared state-of-the-art proposed methods. In short, the proposed modified LSTM technique works great for category detection and sentiment analysis as well.

### 2.3. Hybrid-Based Approaches

HB techniques are the combination of different recommendation system techniques to overcome the drawbacks and limitations of such methods [21]. HB method typically combines the CF and CB methods to mix the benefits of such approaches. To achieve higher performance, HB integrates the profiles or features of users, rating of users, and addresses the issue of CF and CB as well [27]. Here, some of last year's approaches have been discussed. To increase the timeliness and accuracy of the movie, mobile RS, sentiment analysis, and HBRS model are applied on the Apache platform by [28]. In the proposed technique, to obtain the preliminary list, the fusion recommendation method is applied. For enhancing the list, sentiment analysis is applied. In the end, the model is employed on the Apache platform. The results confirm that the proposed method beats the hybrid RS traditional method that integrates CB and CF based on evaluation standards: precision, F-measure, FP rate, and TP rate.

The article [29], proposed a hybrid neural recommendation model to study the representation of items and users deeply from both reviews and ratings. The proposed model contains three main mechanisms such as rating patterns of items and users' explicit features and learn, a deep rating-based encoder for text review, a model of items and user feedback-based encoder, and a prediction component based on items and users review and rating. In addition, for modelling items and users seeing different in formativeness have different reviews, a new mechanism review-level attention included with representation based on rating as a request vector to choose useful feedback. The result shows that this hybrid model beat the existing techniques.

In the research article [30], the authors proposed a monolithic hybrid recommender system named predictor, that integrates recommender components composed of a fuzzy expert system, CF system, and CB system. The proposed method recommends movies. The method works with users unpopular and favorite genres, using fuzzy expert system movies recommended finally list can be determined which estimates the movie's importance. The expert method works with numerous constraints such as the number of ratings, level of resemblance between movies rated already, and movie rating average. Therefore, this method achieves better outcomes than existing methods such as the CB system, weighted hybrid system, and CF systems.  Table 3 shows the existing work of HB.

Data security is one of the main concerns in today's world especially if it is related to health records. The lack of security and confidentiality of clinical health records is one of the main issues. A decentralized Personal Health Record (PHR) can be maintained utilizing the Interplanetary File System (IPFS) [31] to provide patients immediate access to their information. However, IPFS has some issues which can lead to permanent data loss. To overcome this issue, a novel blockchain technology based IPFS architecture is proposed that promotes quicker retrieval and consistent PHR.

Several works have been done in rating prediction. The comparison of item-item and user-user CF algorithms such as user K-NN (cosine similarity), user K-NN (Pearson correlation), item K-NN (cosine similarity) and item K-NN (Pearson correlation) with matrix-based methods such as matrix factorization, biased MF and factor wise MF have not done in existing work. No existing work shows the comparison of all the above algorithms.

## 3. Problem Formulation and Statement

Here, we disuse the problem formulation and problem statement.

### 3.1. Problem Formulation

The proposed work goal or purpose is to predict unknown item ratings. We apply feature vectors to predict rating. We extract a set of items *I*={*i*_1_ … *i* *N*} and a set of users *V*={*v*_1_ … *v* *M*} from datasets. A rating matrix is also set i.e., *R*=[*R*_*v*,*i*_]*M* × *N*, where *R*_*v*,*i*_ presents item to user rating.

The matrix a gives similarity interest values i.e., *S*=[*S*_*u*,*v*_]*M* × *M*, where *S*_*u*,*v*_ represents the resemblance interest between user *v* and user *u*. The matrix a gives similarity values of rating *R*=[*R*_*u*,*v*_]*M* × *M*, where *R*_*u*,*v*_ represents the rating similarity between users *v* and *u*. *B*=[*B*_*u*,*v*_]*M* × *M* represents the rating interpersonal diffusion behaviour between users. *P*=[*P*_*u*,*i*_]*M* × *N* presents the number of personal interests. Our work is to give an item *i* ∈ *I* and user *u* ∈ *V* and *R*_*v*,*i*_ is predicted. By using MF, we calculate the rating prediction of *i* on *u.*

### 3.2. Problem Statement

In current years, one of the hottest topics in the big data field is recommender systems, specifically with the growth and development of the World Wide Web. To improve the process of recommendation, several research studies have been carried out. In the marketing and business area, frequently, our decision depends upon the product or product rating or review and other people's interests or opinions. A higher product rating means more orders and sales. We decide to combine rating prediction and recommendations to predict items that are relevant to the user's interests.

In our proposed work, we intend to predict item-item or user-user rating prediction by applying collaborating filtering techniques on different datasets. We use similarity-based algorithms that reduce the data sparsity problem and help to predict item-item or user-user rating.

## 4. Proposed Research Methodology

In this section, the first section gives the discussion about the proposed framework. The second section discussed about the algorithms. Datasets are discussed in the third section and the last section gives the discussion about the performance evaluation measures used to judge the performance.

### 4.1. Proposed Framework

**Figure 1:**
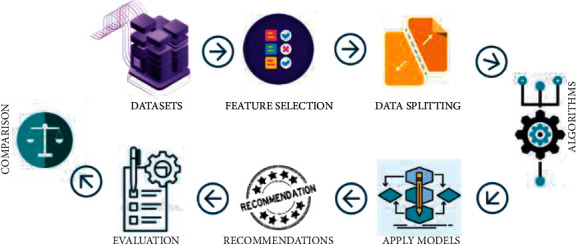
Framework showing steps of the proposed work.

In the proposed research work, firstly we have downloaded three real-world datasets and have selected the required features for empirical analysis. Then, we have applied state-of-the-art CF algorithms with similarity measures and determine the best one by comparing with the widely used algorithms. Then, we have applied the baseline algorithms on our selected datasets and compared the best algorithm with them. Finally, the evaluation measures are used to evaluate the results. In the end, we have compared the results of existing research work with the proposed method.  Figure 1 shows the architecture of our research work.

#### 4.1.1. Features Engineering

Feature engineering shows the features used in the research work for obtaining results.  Table 4 shows the symbols and notations used in this document.

### 4.2. User-User and Item-Item CF Algorithms

Here we explain the user-user and item-item CF algorithms. Followings are the algorithms CF algorithm:

*(1) User-to-User Based CF*. This technique is used to predict products/items that users like based on the rating given by the other user having similar interests. User-to-user similarity is based on the similarity between two users having the same interest by using distance metrics (Euclidean Distance, Cosine similarity, Pearson's correlation, etc.) and then find the similar users and recommended products which similar interest the user rated or liked. Similarity *W*_*u*,*v*_ between two users *v* and *u, W*_*i*,*j*_ between two items *j* and *i*, or is measured by calculating the *Pearson correlation* using the following equation:

(1) $$\begin{array}{c} w_{u,v\;}=\;\frac{\sum i\epsilon I\left(r_{u,i}-\;\underline{r_{u}}\right)\left(r_{v,i}-\;\underline{r_{v}}\right)}{\sqrt{\sum_{i\epsilon I}{\left(r_{u,i}-\;\underline{r_{u}}\right)}^{2}\;}\sqrt{\sum_{i\epsilon I}{\left(r_{v,i}-\;\underline{r_{v}}\right)}^{2}}}. \end{array}$$

By using (2), we will carry out a prediction for the active user i.e., “*a*” on definite item *I* and take the average weighted of all ratings on that item.

(2) $$\begin{array}{c} P_{a,i}=\;\underline{r_{a}}+\;\frac{\sum u\in U\left(r_{u,i}-\;\underline{r_{u}}\right).\;W_{a,u}}{\sum_{u\in U}\left|W_{a,u}\right|}. \end{array}$$

**Figure 2:**
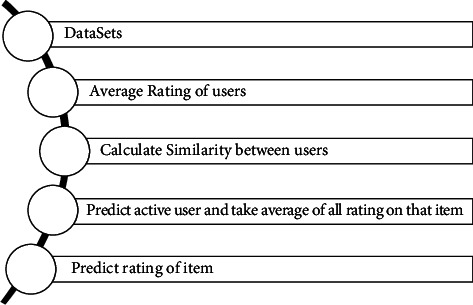
Steps of user-to-user-based collaborating filtering.

We will use the following steps for user-to-user-based similarities and Figure 2 explains these following steps:

1. Load input matrix dataset containing ratings provided by users
2. Find the average rating of all users
3. Then calculate similarity *w*_*u*,*v*_ between two each two set of users' *u* and *v*
4. Predict active user “*a*” with definite item *i* and take all item rating average
5. Predict rating of the item

*(2) Item-to-Item Based CF*. Item-to-item similarity is based on the similarity calculation between items. Based on these similarities, products are recommended to those users who have rated or liked similar products in past. The similarity between two users *i* and *j*, *W*_*i*,*j*_ between two items *i* and *j*, is measured by calculating the Pearson correlation using the following equation:

(3) $$\begin{array}{c} w_{i,j\;}=\;\frac{\sum i\epsilon I\left(r_{u,i}-\;\underline{r_{u}}\right)\left(r_{v,i}-\;\underline{r_{v}}\right)}{\sqrt{\sum_{i\epsilon I}{\left(r_{u,i}-\;\underline{r_{u}}\right)}^{2}}\;\sqrt{\sum_{i\epsilon I}{\left(r_{v,i}-\;\underline{r_{v}}\right)}^{2}}}. \end{array}$$

By using Equation (4), we will carry out the prediction for the active user, i.e., “*a*” on definite item *i* and take the average weighted of all ratings on that item.

(4) $$\begin{array}{c} P_{a,i}=\;\underline{r_{a}}+\;\frac{\sum u\in U\left(r_{u,i}-\;\underline{r_{u}}\right).\;W_{a,u}}{\sum_{u\in U}\left|W_{a,u}\right|}. \end{array}$$

**Figure 3:**
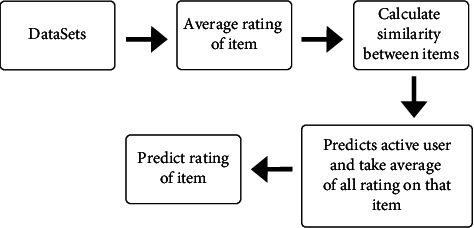
Steps of item-to-item-based collaborating filtering.

We will use the following steps for item-to-item-based similarities and Figure 3 explains these following steps:

1. Take the input of the matrix of ratings of users in the dataset
2. Find the average rating of items
3. Then calculate similarity *w*_*i*,*j*_ between items *i* and *j*
4. Predict active user “*a*” with definite item *i* and take all item rating average
5. Predict rating of the item

#### 4.2.1. Modified Cosine Similarity for Rating Prediction

Cosine similarity is used to calculate the resemblance between items or products. The similarity is calculated between vector angles. By using equation (5), similarities are calculated.

(5) $$\begin{array}{c} \text{sim}\left(\overset{⟶}{u},\overset{⟶}{v}\right)=\frac{\overset{⟶}{u}\cdot \overset{⟶}{v}}{\left|\overset{⟶}{u}\right|*\left|\overset{⟶}{v}\right|}. \end{array}$$

*U*: user's set who have rated items *i* and *j*.

By using Equation (6), the cosine similarity between items is calculated.

(6) $$\begin{array}{c} \text{sim}\left(\overset{⟶}{i},\overset{⟶}{j}\right)=\;\frac{\sum_{u\in U}\left(r_{u,i}-\underline{r_{u}}\right)\left(r_{u,j}-\underline{r_{u}}\right)}{\sqrt{\sum_{u\in U}{\left(r_{u,i}-\underline{r_{u}}\right)}^{2}}\sqrt{\sum_{u\in U}{\left(r_{u,j}-\underline{r_{u}}\right)}^{2}}}. \end{array}$$

#### 4.2.2. Baseline Methods

Here, we explain the baseline method used for comparison. Following are the baseline methods.

*Slope One*. Slope one method is a recommended method based on ratings which works on the deviation of the principle intuitive between user and items. The algorithm used a simple formula that subtracts the two items average only to determine the deviation. Then, the user's rating is used for some items, the deviation used in these rating predictions, and other items rated by users. *f*(*x*)=*x*+*b* is a predictor. *r*_*Bj*_= *r*_*Bi*_+( *r*_*Aj*_ −  *r*_*Ai*_).*r*_*Bj*_ is the rating prediction, user *B* rated item *j*. The prediction process has two steps i.e., (1) find deviation *dev*_*j*,*k*_ between item *k* and item *j*. (2) Find unknown rating *Pu*_*j*_ that means item *j* rated by the target user. Both rate items *k* and *j* are presented by *I*_*jk*_. *R*_*j*_ is presented by the target user. Equations (7) and (8) are shown by the following formula:

(7) $$\begin{array}{c} {\text{dev}}_{j,k}=\sum_{u_{i}\in I_{jk}}\frac{r_{ij}-r_{ik}}{\text{card}\left(I_{jk}\right)}, \end{array}$$

(8) $$\begin{array}{c} P{\left(u\right)}_{j}=\;\frac{\sum_{k\in R_{j}}\left(de\;v_{j,k}+u_{k}\right)}{\text{card}\left(R_{j}\right)}. \end{array}$$

*Random*. A random algorithm is a method in which as a part of the method logic uses the randomness of sources. This method is basically used to decrease the time complexity or running time, space complexity, or memory used in an algorithm standard. Firstly, the algorithm generates a number randomly i.e., r in a range of specified numbers and based on r's values taking decisions.

#### 4.2.3. Matrix-Based Methods

Here, we explain the matrix-based methods. Following are the matrix-based methods.

*Matrix Factorization.* Items and users are mapped to a dimensionality *f* of the joint latent factor space in the MF model, and item-user relations are described as inner products in that space. Every item *i* relates to a vector *q*_*i*_ ∈ *R*^*f*^ and vector *P*_*u*_ ∈ *R*^*f*^ relates to user *u*. The aspects of *q*_*i*_ quantify the extent to which an item possesses certain factors, negative or positive, for a given item. The features of  *P*_*u*_, for a particular user *u*, measure the user's item interest levels that score high on the associated variables, which can be positive or negative. Dot product resulting, *q*_*i*_^*T*^*P*_*u*_ represents the user *u* and item *i* interaction and the whole interest of users in item's features. Equation (9) shows the formula of matrix factorization.

(9) $$\begin{array}{c} {\hat{r}}_{ui}=q_{i}^{T}P_{u}. \end{array}$$

*Biased MF*. On the basis of matrix factorization, the method-based MF [32], which is representing features having explicit rating value by adding a dimensional factor higher that makes the presentation factorized explainable. The method can be used for a variety of discrete features, i.e., age, type, genres, rating, and so on. In Equation (5), *b*_*u*_ represent bias user and for bias item *b*_*i*_ is used. *A* present all items in each feature average to do bias adjustment, where *a*_*c*_ represents the rating average to value type *c*, *b*_*u*_*c* presents bia rating that *u* user type *c* prefer, *b*_*i*_*c* represent the bias popularity of *i* item on *c* type, *C*(*i*) presents the value type set that belongs to *i*, and elements count represented by |*C*(*i*)|. Equation (10) shows the formula of biased MF.

(10) $$\begin{array}{c} \text{bias}=\;\frac{\sum_{c\in C\left(i\right)}a_{c}+b_{u}c+\;b_{i}c}{\left|C\left(i\right)\right|}. \end{array}$$

### 4.3. Datasets

We are taking real-world datasets from movies. In this research study, we have incorporated two benchmark datasets, including MovieLens and CiaoDVD datasets. Both of these datasets are properly balanced and contain real world users' ratings over different movies. The sizes of the same domain datasets are different and contain variations of sparsity and rating ranges. These datasets are famous for evaluation purposes in CF recommender systems:

(i) *The MovieLens Dataset*. The MovieLens dataset consists of two variations: MovieLens 100k (https://grouplens.org/datasets/movielens/100k/ Accessed on May 03, 2021.) ratings and MovieLens 1M (https://grouplens.org/datasets/movielens/1m/ Accessed on May 03, 2021.) ratings. In the dataset of 100k ratings, as the names indicate, 100,000 ratings have been collected from 943 users on 1682 different movies. The range of ratings varies from 1 to 5. Approximately 20 movies have been rated by each user. In the dataset of 1M ratings, 6040 users of the MovieLens platform rated anonymously almost 3900 movies. These datasets are the benchmarks used in many kinds of research.
(ii) *The CiaoDVD Dataset*. The CiaoDVD (http://konect.cc/networks/librec-ciaodvd-movie_ratings/ Accessed on May 03, 2021.) dataset is a newly collected dataset of the movie domain. The dataset contains 17615 users that have rated 16121 items (movies), and the total ratings are about 700k. The sparsity of the dataset is almost 99.62%. Values of ratings in this dataset are like MovieLens dataset *i* − *e* 1 (worst) to 5 (best).

### 4.4. Performance Evaluation Measures

It is used to evaluate the research work. Here, the performance evaluation measures will be discussed in detail:

(i) *Root Mean Square Error*. RMSE is used to calculate the difference between the values observed and the values predicted. By using Equation (11) to calculate RMSE and *q* is predicted, and *r* is a known value.
  (11) $$\begin{array}{c} \text{RMSE}=\sqrt{\frac{\sum_{i=1}^{m}{\left(q-r\right)}^{2}}{m}}. \end{array}$$
(ii) *Mean Absolute Error*. MAE is used to calculate the difference between predicted and true values. By using equation (12) to calculate this error. *m* is the number of errors, *y* is prediction, and *x* is the true value.

(12) $$\begin{array}{c} \text{MAE}=\frac{1}{m}\sum_{i=1}^{m}\left|y_{i}-x\right|. \end{array}$$

## 5. Results and Discussion

Let us discuss here the experimental results using three real world datasets, i.e., MovieLens 100k, MovieLens 1M, and CiaoDVD. In this research work, a comparison of user-user and item-item CF algorithms, i.e., user K-NN (cosine similarity), user K-NN (Pearson correlation), item K-NN (cosine similarity), and item K-NN (Pearson correlation) is performed, and the best algorithm is identified.

Later on, the best algorithms are compared with matrix-based methods, i.e., matrix factorization, biased MF, and factor wise MF. In addition, the best algorithm is further compared with baseline methods including slope one, random, global average, and user item baseline. Then, all methods including CF, MF, and baseline are compared and identified the best algorithm based on performance evaluation measures. Finally, we also compare the best algorithm with existing methods.

### 5.1. Comparison of User-User and Item-Item CF

**Figure 4:**
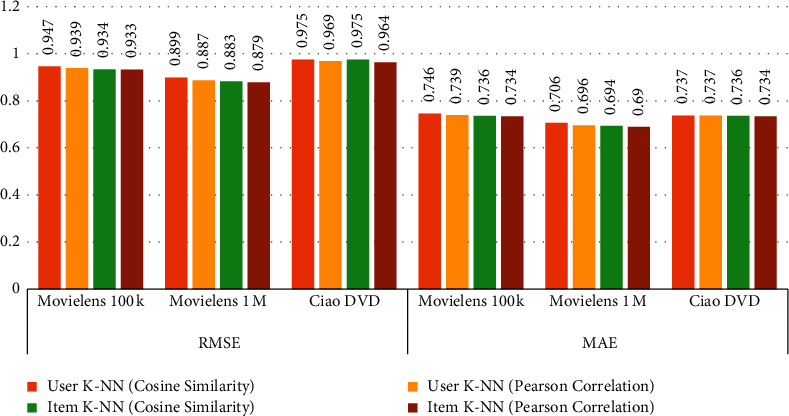
Comparison of user-user and item-item CF.

The user-user and item-item CF algorithms are applied on the selected datasets and the results are computed as presented in Table 5. The datasets are divided into 70% and 30% for training and testing, respectively, and select feature *User-Id* set as a user identification and *ratings* set as the prediction. To compute RMSE and MAE values for MovieLens 100k, MovieLens 1M, and CiaoDVD datasets, user K-NN (cosine similarity), User K-NN (Pearson correlation), item K-NN (cosine similarity), and item K-NN (Pearson correlation) are applied. According to the computed results, the item K-NN (Pearson correlation) gives efficient RMSE and MAE compared to other CF methods.  Figure 4 can also explain the comparison of collected outcomes.

### 5.2. Comparison of Matrix-Based Methods with Item-Item (Pearson Correlation)

**Figure 5:**
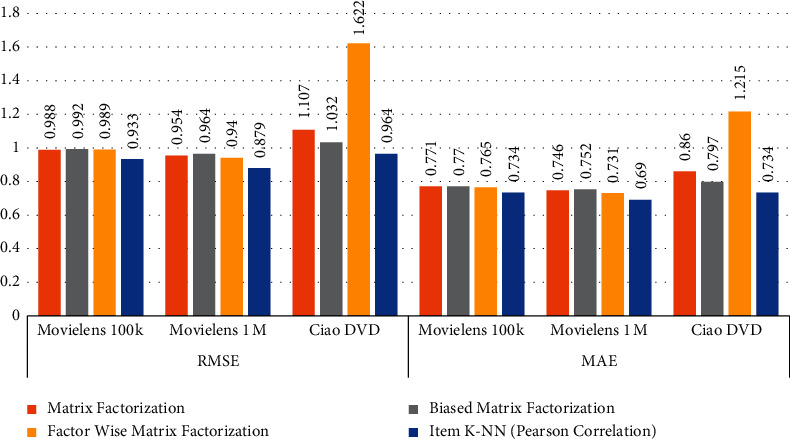
Comparison of MF methods with Item-Item (Pearson Correlation) method.

The matrix-based methods are applied on the selected datasets and the results are computed as presented in Table 6. The datasets are divided into 70% and 30% for training and testing, respectively, and select features *User-Id* set as a user identification and *Ratings* set as the prediction. To compute RMSE and MAE values for MovieLens 100k, MovieLens 1M, and CiaoDVD datasets, Matrix-Based Methods such as matrix factorization, biased MF, and factor wise MF are applied. In addition, compare the matrix-based method outcomes with item K-NN (Pearson correlation). According to the computed results, the item K-NN (Pearson correlation) gives efficient RMSE and MAE compared to matrix-based methods.  Figure 5 can also explains the comparison of collected outcomes.

### 5.3. Comparison of Baseline Methods with Item-Item (Pearson Correlation)

**Figure 6:**
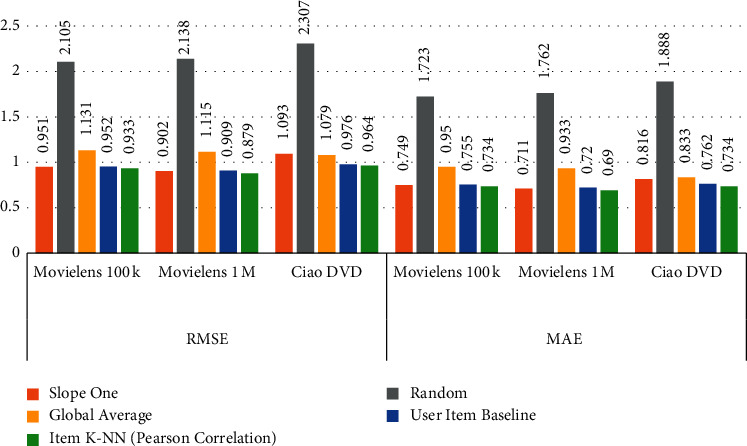
Comparison of BL methods with Item-Item (Pearson Correlation) method.

The Baseline Methods are applied on the selected datasets and the results are computed as presented in Table 7. The datasets are divided into 70% and 30% for training and testing, respectively, and select features *User-Id* set as a user identification and *Ratings* set as the prediction. To compute RMSE and MAE values for MovieLens 100k, MovieLens 1M, and CiaoDVD datasets, Slope One, Random, Global Average, and User Item Baseline are applied. In addition, compare the Baseline Method outcomes with item K-NN (Pearson correlation). According to the computed results, the item K-NN (Pearson correlation) gives efficient RMSE and MAE compared to Baseline Methods.  Figure 6 can also explains the comparison of collected outcomes.

### 5.4. Comparison of All Methods Applied to the Above-Mentioned Datasets as a Whole

**Figure 7:**
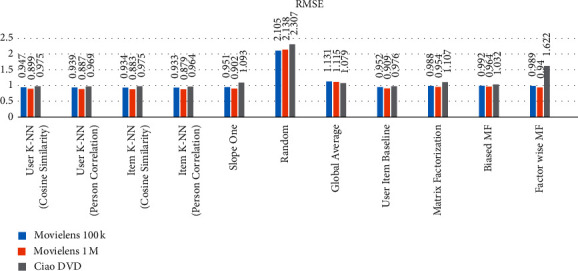
Comparison of Applied Algorithms on Datasets RMSE values.

**Figure 8:**
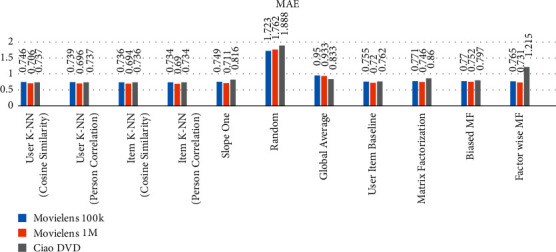
Comparison of Applied Algorithms on Dataset MAE values.

The comparison of user-user and item-item CF algorithms, matrix-based methods, and baseline method are applied on the selected datasets and the results are computed as presented in Table 8. The datasets are divided into 70% and 30% for training and testing, respectively, and select features *User-Id* set as a user identification and *Ratings* set as the prediction. According to the computed results, the item K-NN (Pearson correlation) gives efficient RMSE and MAE compared to the other CF, matrix-based and baseline methods. Figures 7 and 8 also explain the comparison of collected outcomes.

### 5.5. Comparison of Proposed Method (Item K-NN Pearson Correlation) with Previous Methods

**Figure 9:**
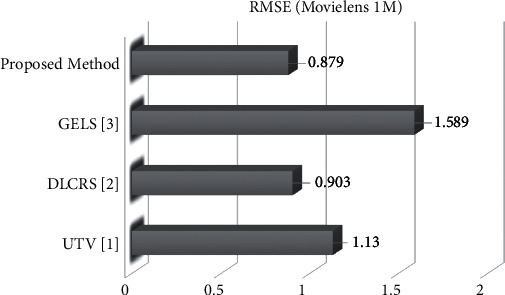
Rmse evaluation comparison with proposed and previous paper method with ML (1M).

At the end item, K-NN (Pearson correlation) is also compared with previously existing methods, i.e., UTV [33], DLCRS [14], and GELS [34]. UTV is a User Triple Vector. This vector uses different component similarity and predicts recommender results. DLCRS is a deep learning technique of collaborative RS. DLCRS method matches both movies and users in the same dimensionality and obtains the latent factor. This method used a logistic sigmoid function for prediction. GELS is a Gravitational Emulation Local Search. This method considers the galaxy as a search space and imagines every problem solution in the search space as an object. The RMSE achieved values of UTV [31], DLCRS [32], and GELS [33] to applying on MovieLens 1M dataset are 1.130, 0903, and 1.587. The item K-NN (Pearson correlation) RMSE value to applying on MovieLens 1M dataset is 0.897, which shows that the item K-NN (Pearson correlation) gives efficient outcomes compared to the previously existing methods.  Figure 9 and Table 9 also explain the comparison of collected outcomes.

The results and discussion may be presented separately or in one combined section, and may optionally be divided into headed subsections.

## 6. Conclusion and Future Work

In this research work, we predict the users' ratings for various items, which are an active research area in collaborative filtering. In this work, we explore various similarity measures based on user-user and item-item rating predictions on different datasets by applying collaborative filtering approaches. The comparison of item-item and user-user CF algorithms, i.e., user K-NN (cosine similarity), user K-NN (Pearson correlation), and item K-NN (cosine similarity) with the proposed method item K-NN (Pearson correlation) and matric based methods with item-item i.e., matrix factorization, biased MF, and factor wise MF with proposed method comparison are carried out. In addition, we do the comparison of baseline methods included with item-item including slope one, random, global average, and user item baseline with the proposed method and comparison of the proposed method with all methods above mentioned. Then, in the end, the proposed method's performance is compared with existing methods. The result shows that the proposed methods give an effective outcome compared to other methods.

Regarding RS, the potential research, future work can be exploring deep learning based latest algorithms such as Recurrent neural networks (RNNs), Convolutional Neural Network (CNN), and Long short-term memory (LSTM) and their variation for the prediction of user's rating. In addition, the hybrid approach can also be explored, which may consider combining context-based approaches with collaborative filtering approaches.

## Figures and Tables

**Table 1:** Showing the comparison of previous work of CF.

Year refer.	Method	Dataset	Results
2020 [14]	Proposed collaborative recommendation system of deep learning method (DLCRS)	MovieLens 1M Movielens 100k	MovieLens 1M: RMSE0.903
2020 [15]	Novel technique that combines similarity measurement based on ranking and similarity measurement based on the structure	MovieLens 1M Movielens 100k	RMSE 0.909 MAE 0.708
2020 [16]	Similarity measure multifactor	CiaoDVD MovieLens 100k FilmTrust	RMSE 1.0084 MAE 0.7835
2021 [17]	Proposed a simple linear model named UserReg, based on the matrix factorization (MF)	MovieLens, FilmTrust, and Yelp	RMSE 0.789
2021 [18]	Proposed the *α*-divergence based on item similarity measures	MovieLens, FilmTrust	MAE 0.74 RMSE 0.97

**Table 2:** Comparison of existing works of content-based filtering.

Year refer.	Method	Dataset	Results
2018 [24]	E-learning recommender system utilizing negative rating	5 groups of students (each have 25 students)	F 35.381
2019 [22]	Semantic web mining approach for recommender system	Web textual dataset	Increases 5.2% accuracy
2019 [23]	Traditional recommender system context by using content based and link stream features	Goodreads MovieLens 20M	RMSE 0.8095
2021 [25]	Content-based group recommendation systems (CB-GRS)	MovieLens 100K HetRec	Precision metric 0.5167

**Table 3:** Comparison of existing work of hybrid-based methods.

Year refer.	Method	Dataset	Results
2018 [28]	Sentiment enhanced recommender model	MovieLens	TP rate 0.645
2019 [29]	Hybrid neural recommendation model	Video games gourmet food	RMSE 1.011
		Yelp 2013	
		Yelp 2014	
2020 [30]	Proposed a monolithic hybrid recommender system named predictor	MovieLens	Precision 81%

**Table 4:** List of symbols and notations used in the document.

Symbols	Description
*u*, *v*	Users
*i*, *j*	Items
*W*_*u*,*v*_	Weight of similarity between two users
*i* ∈ *I*	Summations of items rated by both the users *v* and *u*
*R*	Rating
*r*_*u*,*i*_, *r*_*v*,*i*_	Rating of the user to the item
$\underline{r_{u}},\;\underline{r_{v}}$	Average rating of user
*A*	Active user
*P*_*a*,*i*_	Prediction of the active user to the item
$\underline{r_{a}}$	Active user average rating
*u* ∈ *U*	Summations of user rated both the users *v* and *u*
*W*_*a*,*u*_	Similarity between the weight of active user and user
*R*_*u*_, *R*_*v*_	Rating value of user *u* and *v*

**Table 5:** Comparison of user KNN and item KNN.

Algorithms	RMSE			MAE		
	ML	ML	Ciao DVD	ML	ML	Ciao DVD
	100k	1M		100k	1M	
User K-NN (cosine similarity)	0.947	0.899	0.975	0.746	0.706	0.737
User K-NN (Pearson correlation)	0.939	0.887	0.969	0.739	0.696	0.737
Item K-NN (cosine similarity)	0.934	0.883	0.975	0.736	0.694	0.736
Item K-NN (Pearson correlation)	0.933	0.879	0.964	0.734	0.690	0.734

**Table 6:** Comparison of MF methods with Item-Item (Pearson Correlation) method.

Algorithms	RMSE			MAE			
	ML	ML	Ciao DVD	ML	ML		Ciao DVD
	100k	1M		100k	1M		
Matrix factorization	0.988	0.954	1.107	0.771	0.746		0.860
Biased matrix factorization	0.992	0.964	1.032	0.770	0.752		0.797
Factor wise matrix factorization	0.989	0.940	1.622	0.765	0.731		1.215
Item K-NN (Pearson correlation)	0.933	0.879	0.964	0.734	0.690		0.734

**Table 7:** Comparison of BL methods with Item-Item (Pearson Correlation) method.

Algorithms	RMSE			MAE		
	ML	ML	Ciao DVD	ML 100k	ML	Ciao DVD
	100k	1M			1M	
Slope one	0.951	0.902	1.093	0.749	0.711	0.816
Random	2.105	2.138	2.307	1.723	1.762	1.888
Global average	1.131	1.115	1.079	0.950	0.933	0.833
User item baseline	0.952	0.909	0.976	0.755	0.720	0.762
Item K-NN (Pearson correlation)	0.933	0.879	0.964	0.734	0.690	0.734

**Table 8:** Comparison of Applied Algorithms on Datasets as a whole.

Algorithms	RMSE			MAE		
	ML 100k	ML 1M	Ciao DVD	ML	ML 1M	Ciao DVD
				100k		
User K-NN (cosine similarity)	0.947	0.899	0.975	0.746	0.706	0.737
User K-NN (Pearson correlation)	0.939	0.887	0.969	0.739	0.696	0.737
Item K-NN (cosine similarity)	0.934	0.883	0.975	0.736	0.694	0.736
Item K-NN (Pearson similarity)	**0.933**	**0.879**	**0.964**	**0.734**	**0.690**	**0.734**
Slope one	0.951	0.902	1.093	0.749	0.711	0.816
Random	2.105	2.138	2.307	1.723	1.762	1.888
Global average	1.131	1.115	1.079	0.950	0.933	0.833
User item baseline	0.952	0.909	0.976	0.755	0.720	0.762
Matrix factorization	0.988	0.954	1.107	0.771	0.746	0.860
Biased MF	0.992	0.964	1.032	0.770	0.752	0.797
Factor wise MF	0.989	0.940	1.622	0.765	0.731	1.215

**Table 9:** RMSE evaluation comparison with proposed and previous paper method with ML (1M).

Algorithms	Datasets
	MovieLens 1M
UTV [33]	1.130
DLCRS [14]	0.903
GELS [34]	1.589
Proposed method	0.879

## Data Availability

The datasets used to pursue the findings of this article are freely available online. The URLs of the datasets have been provided in the footnotes in Section 5.1 of Section 5 named as “Experimental Setup.”
